# Supplementary material for: Spaceflight affects neuronal morphology and alters transcellular degradation of neuronal debris in adult Caenorhabditis elegans
Source: iScience. 2021 Jan 29;24(2):102105. doi: 10.1016/j.isci.2021.102105 (PMC7890410; doi:10.1016/j.isci.2021.102105)
Supplement: Document S1. Transparent methods and Figures S1–S4 [file mmc1.pdf]

iScience, Volume *24*

Supplemental Information

Spaceflight affects neuronal morphology and alters transcellular degradation of neuronal debris in adult *Caenorhabditis elegans*

Ricardo Laranjeiro, Girish Harinath, Amelia K. Pollard, Christopher J. Gaffney, Colleen S. Deane, Siva A. Vanapalli, Timothy Etheridge, Nathaniel J. Szewczyk, and Monica Driscoll

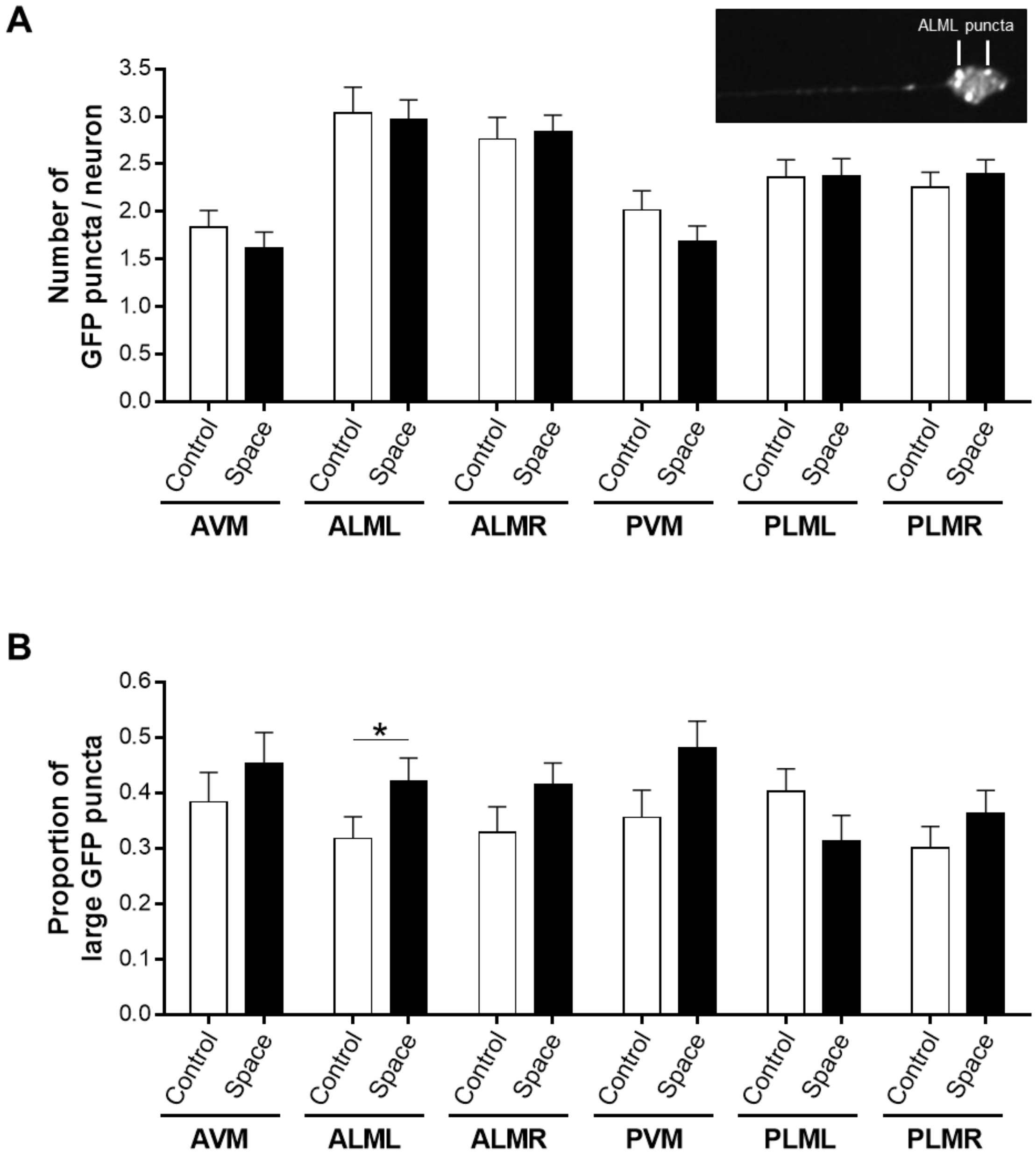

Figure S1. Spaceflight modulates the size of high-intensity GFP fluorescent puncta in touch receptor neurons. Related to Figure 4. (**A**) Number of GFP puncta per cell body in each of the six touch receptor neurons in ground control and spaceflight $P_{mec\text{-}4}GFP$ animals. We include a confocal image from a $P_{mec\text{-}4}GFP$ animal showing GFP puncta in an ALML cell body. Number of touch receptor neurons used for analysis: $n_{Control}$ = 54-61, n_{Space} = 60-64. We determined statistical significance by unpaired two-tailed Student's *t* test. (**B**) Proportion of large GFP puncta per cell body in each of the six touch receptor neurons in ground control and spaceflight $P_{mec\text{-}4}GFP$ animals. We scored GFP puncta as 'large' when diameter ≥ 0.9 μm. Number of touch receptor neurons used for analysis: $n_{Control}$ = 54-61, n_{Space} = 60-64. We determined statistical significance by Fisher's exact test. *$P \leq 0.05$.

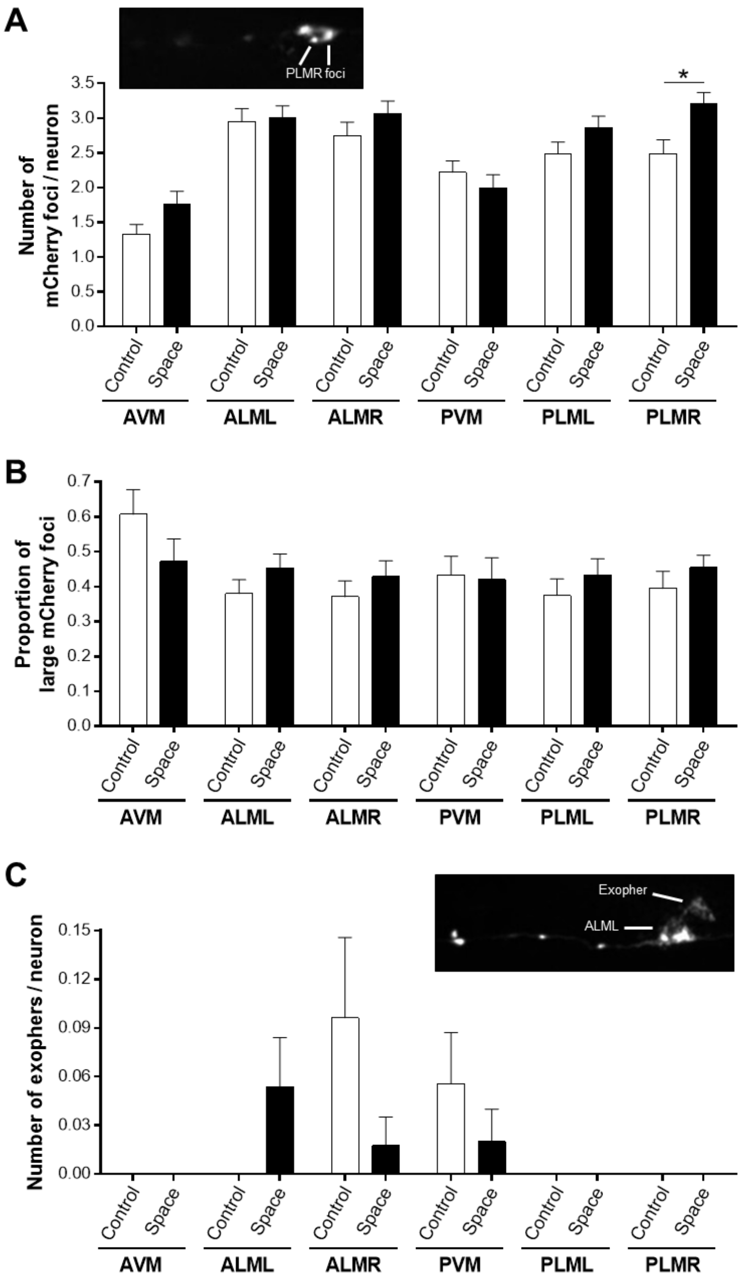

Figure S2. Spaceflight modulates the number of mCherry foci in touch receptor neurons. Related to Figure 5. (**A**) Number of mCherry foci per cell body in each of the six touch receptor neurons in ground control and spaceflight $P_{mec\text{-}4}$*mCherry1* animals. We include a confocal image from a $P_{mec\text{-}4}$*mCherry1* animal showing mCherry foci in a PLMR cell body. Number of touch receptor neurons used for analysis: n_{Control} = 42-61, n_{Space} = 42-66. We determined statistical significance by unpaired two-tailed Student's *t* test. (**B**) Proportion of large mCherry foci per cell body in each of the six touch receptor neurons in ground control and spaceflight $P_{mec\text{-}4}$*mCherry1* animals. We scored mCherry foci as 'large' when diameter ≥ 0.9 µm. Number of touch receptor neurons used for analysis: n_{Control} = 42-61, n_{Space} = 42-66. We determined statistical significance by Fisher's exact test. (**C**) Number of exophers per each of the six touch receptor neurons in ground control and spaceflight $P_{mec\text{-}4}$*mCherry1* animals. We include a confocal image from a $P_{mec\text{-}4}$*mCherry1* animal showing an ALML cell body extruding an exopher. *$P \leq 0.05$.

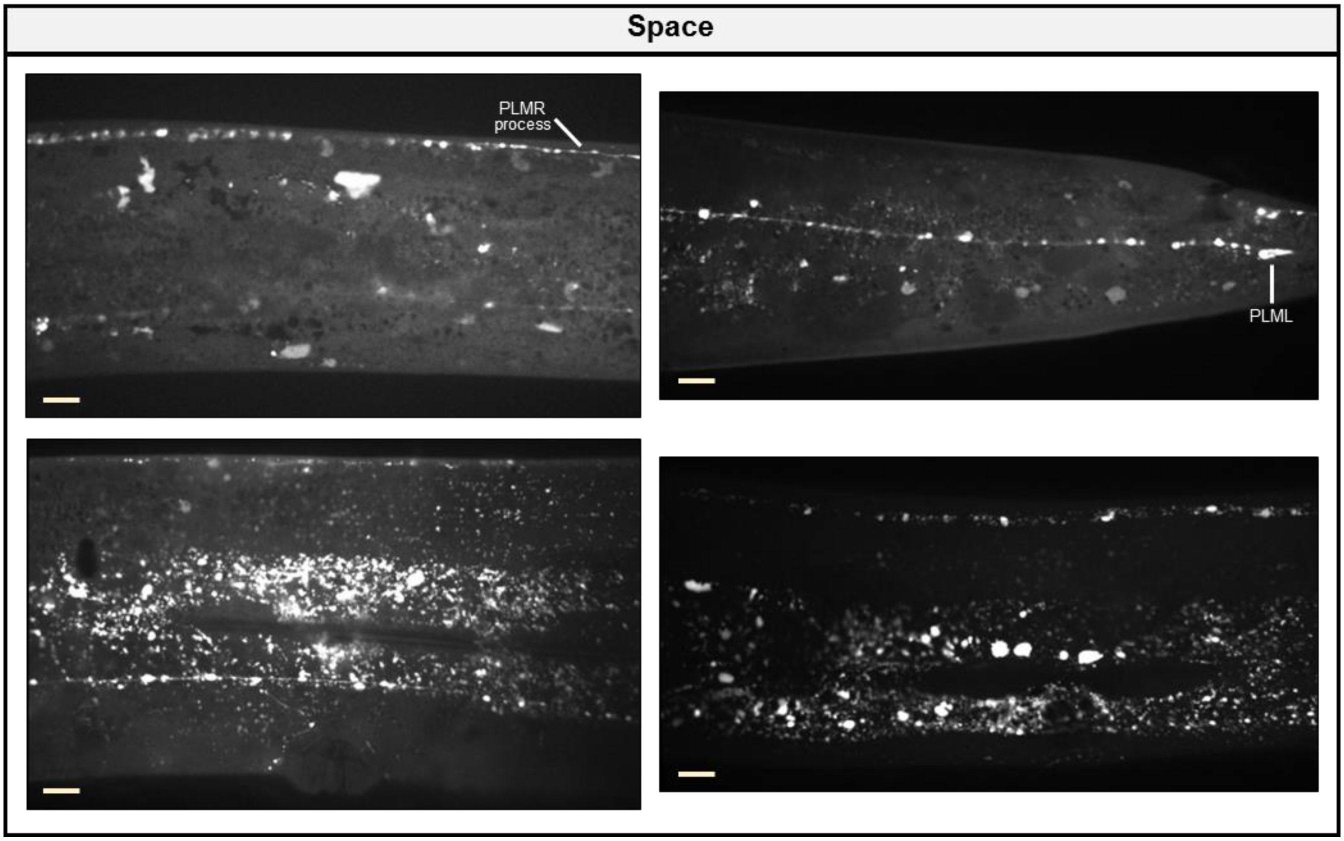

Figure S3. Spaceflight leads to accumulation of neuronal-derived mCherry throughout the body of middle-aged nematodes. Related to Figure 5. Additional representative confocal images of spaceflight *P*$_{mec\text{-}4}$*mCherry1* animals. Scale bars, 10 µm.

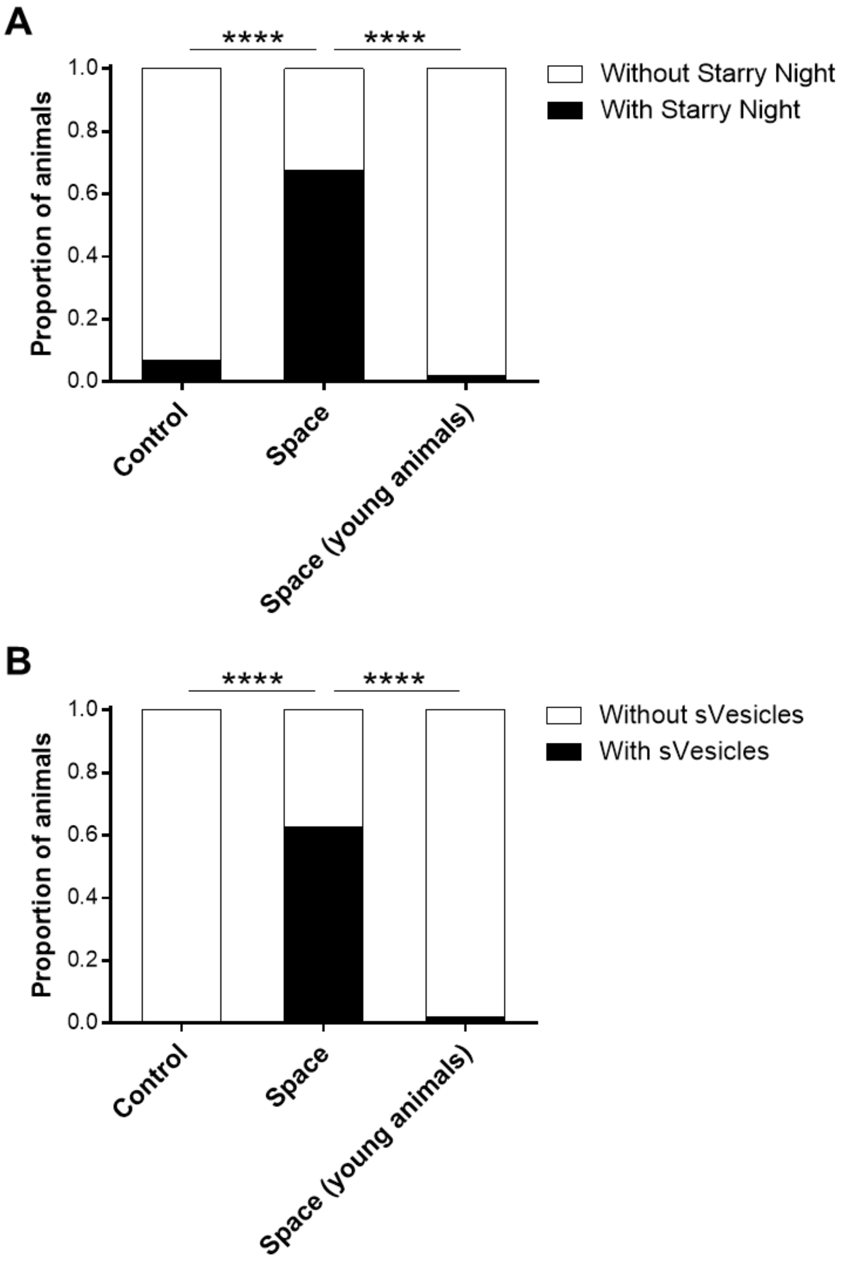

Figure S4. Accumulation of neuronal-derived mCherry throughout the body of space-flown animals appears to be age-dependent. Related to Figure 6. (**A, B**) Proportion of ground control, spaceflight, and young spaceflight $P_{mec\text{-}4}$*mCherry1* animals with/without Starry Night (**A**) and with/without sVesicles (**B**). Note that we obtained young adults from the same spaceflight bag as the middle-aged adults given that some progeny were able to escape FUdR inhibition to develop in the presence of FUdR. We easily identified the spaceflight young adults from the middle-aged adults by their reduced size and we randomly selected 50 young adults for scoring. Number of animals used for analysis: n_{Control} = 72, n_{Space} = 83, $n_{\text{Space (young animals)}}$ = 50. We determined statistical significance by Fisher's exact test. ****$P \leq 0.0001$.

Transparent Methods

C. elegans strains and maintenance

The *C. elegans* strains used in this study were: MF190 *hmIs4[des-2::GFP + rol-6(su1006)]* (Oren-Suissa et al., 2010), ZB4510 *zdIs5[$P_{mec\text{-}4}$GFP + lin-15(+)] I*, and ZB4065 *bzIs166[$P_{mec\text{-}4}$mCherry1]* (Melentijevic et al., 2017). ZB4510 was generated by outcrossing SK4005 *zdIs5[$P_{mec\text{-}4}$GFP + lin-15(+)] I* (Clark and Chiu, 2003) to N2 five times. Approximately two weeks prior to the launch procedure, we transferred animals from NGM agar plates seeded with live *Escherichia coli* OP50-1 to a liquid culture of S-Basal with freeze-dried *Escherichia coli* OP50 (LabTIE, 1 vial per 250 mL of S-Basal). After this point, we maintained all *C. elegans* strains at 20°C in the liquid culture in 75 cm^2 cell culture flasks (Pollard et al., 2020).

Sample preparation for spaceflight

We synchronized *C. elegans* populations by bleaching (20% bleach in 2N NaOH) to obtain eggs or by gravity settling (Gaffney et al., 2014) to obtain L1 larvae. When the synchronized animals reached L4/young adult stage, we moved approximately 300 animals per strain to 6-well tissue culture dishes in 1.2 mL of S-Basal with freeze-dried OP50 and 400 µM FUdR. After an overnight incubation at 20°C, we added 5.3 mL of S-Basal with freeze-dried OP50 and 400 µM FUdR to each well and loaded the entire volume (6.5 mL) into PE flight culture bags (Pollard et al., 2020) on December 2, 2018. Culture bags were then placed into experiment cassettes (ECs, Kayser Italia) (Pollard et al., 2020) to provide an additional level of containment and to protect the *C. elegans* cultures from launch and/or in-flight damage. Our samples were then kept in cold stowage (8-13°C) until they reached the ISS. Launch of SpaceX CRS-16 occurred on December 5, 2018 and docking to the ISS occurred on December 8, 2018. On December 9, 2018, our *C. elegans* samples were transferred to 20°C for five days on the ISS. After the five days, samples were transferred to −80°C and kept frozen until return to Earth.

For ground control samples, we performed the same synchronization protocol as for spaceflight samples and we loaded *C. elegans* into the PE flight culture bags on December 5, 2018. We also placed ground control bags into ECs and exposed them to the same time frame/temperatures as spaceflight samples but we maintained cultures always on Earth. Temperature conditions experienced by spaceflight samples were recorded by iButton digital thermometers (iButtonLink) placed inside ECs and allowed us to expose ground control

samples to similar conditions. We deposited detailed temperature recordings from spaceflight and control samples in the Harvard Dataverse repository (https://doi.org/10.7910/DVN/ATOJCJ).

Confocal microscopy

We stored the frozen PE flight culture bags at −80°C until we were ready to start the confocal imaging. We thawed small fragments of each culture bag in 2% PFA/M9 buffer for approximately 30 min at room temperature. Based on previous testing, we determined that thawing *C. elegans* while simultaneously fixing them in PFA was essential to maintain a strong fluorescence signal in the reporter strains used in this study. After thaw/fixation, we carefully selected, under a stereo microscope, the middle-aged adults present in the sample, which we could easily identify from young animals by their large size. We stored animals in M9 buffer at 4°C until imaging. We performed confocal imaging with an X-Light V2 TP spinning disk unit (CrestOptics) mounted to an Axio Observer.Z1 microscope (ZEISS) using MetaMorph Premier software (Molecular Devices). We scored all middle-aged adults that we recovered from the frozen culture bags, except to score the number of beads/bubbles in PVD dendrites and the location of Starry Night and sVesicles in the anterior-posterior body axis and in different tissues, for which we randomly selected a subgroup of middle-aged animals. For PVD neurons, we first acquired a series of z-stack images from the region anterior to the PVD soma and then obtained the maximum intensity projection image using ImageJ, in which we scored PVD dendritic morphologies. For touch receptor neurons, we scored phenotypes directly on the confocal microscope and we took representative images and z-stack series.

Whole-mount LMP-1 antibody staining

After thawing/fixing $P_{mec\text{-}4}$*mCherry1* animals in 2% PFA/M9 buffer for 30 min at room temperature, we tried different permeabilization methods prior to anti-LMP-1 antibody incubation. We permeabilized animals in 1% Triton X-100/M9 buffer for 45 min, in 1% glacial acetic acid/ethanol for 30 min, or at −80°C in methanol for 1 hour then in acetone for 30 min followed by a serial rehydration at room temperature in 75%, 50%, 25%, and 0% methanol in TBS (100mM NaCl, 50mM Tris-HCl, pH7.5) (Djeddi et al., 2015). We washed samples with 0.1% Tween-20/M9 buffer followed by blocking in 2% BSA/0.1% Tween-20/M9 buffer for 1 hour at room temperature. We incubated samples with anti-LMP-1 antibody (1D4B, Developmental

Studies Hybridoma Bank, 1:5 or 1:10 dilution of supernatant) (Hadwiger et al., 2010) in blocking solution overnight at 4°C. After several washes with 0.1% Tween-20/M9 buffer, we incubated samples with 1:1000 or 1:2000 secondary antibody Goat anti-Mouse, Alexa Fluor 488 (Invitrogen) in blocking solution at room temperature. After washing thoroughly with 0.1% Tween-20/M9 buffer, we imaged samples in the confocal microscope. Despite the different permeabilization methods and antibody concentrations tested, we were unable to observe specific LMP-1 staining in $P_{mec\text{-}4}$*mCherry1* animals.

Statistical analyses

The data in this study are presented as the mean ± standard error of the mean (SEM). The specific number of data points and the test used for each statistical analysis are presented in the figure legends.

Supplemental References

Clark, S.G., and Chiu, C. (2003). C. elegans ZAG-1, a Zn-finger-homeodomain protein, regulates axonal development and neuronal differentiation. Development *130*, 3781-3794.

Djeddi, A., Al Rawi, S., Deuve, J.L., Perrois, C., Liu, Y.Y., Russeau, M., Sachse, M., and Galy, V. (2015). Sperm-inherited organelle clearance in C. elegans relies on LC3-dependent autophagosome targeting to the pericentrosomal area. Development *142*, 1705-1716.

Gaffney, C.J., Bass, J.J., Barratt, T.F., and Szewczyk, N.J. (2014). Methods to assess subcellular compartments of muscle in C. elegans. J Vis Exp, e52043.

Hadwiger, G., Dour, S., Arur, S., Fox, P., and Nonet, M.L. (2010). A monoclonal antibody toolkit for C. elegans. PloS one *5*, e10161.

Melentijevic, I., Toth, M.L., Arnold, M.L., Guasp, R.J., Harinath, G., Nguyen, K.C., Taub, D., Parker, J.A., Neri, C., Gabel, C.V.*, et al.* (2017). C. elegans neurons jettison protein aggregates and mitochondria under neurotoxic stress. Nature *542*, 367-371.

Oren-Suissa, M., Hall, D.H., Treinin, M., Shemer, G., and Podbilewicz, B. (2010). The fusogen EFF-1 controls sculpting of mechanosensory dendrites. Science *328*, 1285-1288.

Pollard, A.K., Gaffney, C.J., Deane, C.S., Balsamo, M., Cooke, M., Ellwood, R.A., Hewitt, J.E., Mierzwa, B.E., Mariani, A., Vanapalli, S.A.*, et al.* (2020). Molecular Muscle Experiment: Hardware and Operational Lessons for Future Astrobiology Space Experiments. Astrobiology.
